# Drug-Resistant Juvenile Myoclonic Epilepsy: Misdiagnosis of Progressive Myoclonus Epilepsy

**DOI:** 10.3389/fneur.2019.00946

**Published:** 2019-09-10

**Authors:** Sarah Martin, Adam Strzelczyk, Silvia Lindlar, Kristina Krause, Philipp S. Reif, Katja Menzler, Andreas G. Chiocchetti, Felix Rosenow, Susanne Knake, Karl Martin Klein

**Affiliations:** ^1^Center for Personalized Translational Epilepsy Research (CePTER), Frankfurt am Main, Germany; ^2^Epilepsy Center Hessen, Department of Neurology, Philipps University Marburg, Marburg, Germany; ^3^Epilepsy Center Frankfurt Rhine-Main, Department of Neurology, Goethe University, Frankfurt am Main, Germany; ^4^Department of Child and Adolescent Psychiatry, Psychosomatics and Psychotherapy, Goethe University, Frankfurt am Main, Germany; ^5^Departments of Clinical Neurosciences, Medical Genetics and Community Health Sciences, Hotchkiss Brain Institute, Alberta Children's Hospital Research Institute, Cumming School of Medicine, University of Calgary, Calgary, AB, Canada

**Keywords:** epilepsy, genetics, Lafora disease, progressive myoclonus epilepsy, juvenile myoclonic epilepsy, pharmacoresistance

## Abstract

Juvenile myoclonic epilepsy (JME) is a common epilepsy syndrome characterized by bilateral myoclonic and tonic-clonic seizures typically starting in adolescence and responding well to medication. Misdiagnosis of a more severe progressive myoclonus epilepsy (PME) as JME has been suggested as a cause of drug-resistance. Medical records of the Epilepsy Center Hessen-Marburg between 2005 and 2014 were automatically selected using keywords and manually reviewed regarding the presence of a JME diagnosis at any timepoint. The identified patients were evaluated regarding seizure outcome and drug resistance according to ILAE criteria. 87/168 identified JME patients were seizure-free at last follow-up including 61 drug-responsive patients (group NDR). Seventy-eight patients were not seizure-free including 26 drug-resistant patients (group DR). Valproate was the most efficacious AED. The JME diagnosis was revised in 7 patients of group DR including 6 in whom the diagnosis had already been questioned or revised during clinical follow-up. One of these was finally diagnosed with PME (genetically confirmed Lafora disease) based on genetic testing. She was initially reviewed at age 29 yrs and considered to be inconsistent with PME. Intellectual disability (*p* = 0.025), cognitive impairment (*p* < 0.001), febrile seizures in first-degree relatives (*p* = 0.023) and prominent dialeptic seizures (*p* = 0.009) where significantly more frequent in group DR. Individuals with PME are rarely found among drug-resistant alleged JME patients in a tertiary epilepsy center. Even a very detailed review by experienced epileptologists may not identify the presence of PME before the typical features evolve underpinning the need for early genetic testing in drug-resistant JME patients.

## Introduction

Juvenile myoclonic epilepsy (JME) is a common epilepsy syndrome occurring in 5–10% of all epilepsies (1, 2). It is characterized by onset of bilateral myoclonic seizures (MS) and bilateral tonic-clonic seizures (BTCS) between age 8 and 25 years with normal development and cognition (ILAE commission on classification and terminology)^1^. Additional dialeptic seizures (DS) occur in 30% (2, 3). Typical triggers for seizures are sleep deprivation and alcohol intake. The EEG hallmark is generalized spike-wave complexes with frequencies around 3.5–6 Hz and normal background. JME is a subtype of genetic (previously idiopathic) generalized epilepsy (GGE). Sometimes evolution from other genetic generalized epilepsy syndromes such as childhood absence epilepsy is seen (4). Although JME typically persist during life, it generally responds well to antiepileptic medication. However, 15–30% of patients are drug resistant (5–7). Misdiagnosis of progressive myoclonus epilepsy as juvenile myoclonic epilepsy has been described as a potential cause for drug resistance (8–11).

Progressive myoclonus epilepsies (PME) are a heterogeneous group of epilepsies characterized by the occurrence of MS associated with variable degrees of cognitive deterioration and ataxia (10, 12). BTCS can occur as well. Similarly to JME, EEG shows generalized spike wave activity. However, there is slowing of the background and progressive clinical deterioration during the course of the disease.

We aimed to clarify if misdiagnosis of PME as JME is a cause for drug resistance in the patient population of a tertiary epilepsy center in Germany and to identify phenotypic differences between drug-responsive and drug-resistant JME patients.

## Methods

### Patient Selection

**Figure 1:**
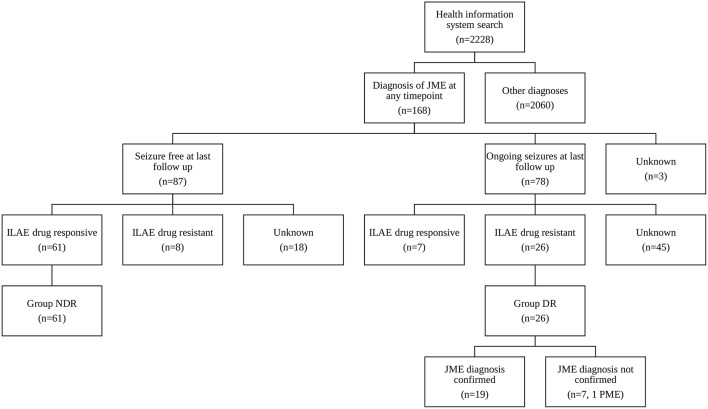
Consort diagram outlining the search strategy and assignment of groups DR and NDR.

At the Epilepsy Center Hessen-Marburg, Department of Neurology, Philipps University Marburg medical reports are written for every inpatient or outpatient visit. The five-dimensional patient-oriented epilepsy classification (13, 14) is a mandatory part of every letter since its introduction. Using the electronic health information system we selected all patients presenting between 2005 and 2014, whose letters included at least one of the search terms “myoclon”, “Myoklon”, or “Janz”. The electronic record of the identified patients was then manually reviewed regarding the presence of a JME diagnosis at any time point (Figure 1). We also included patients with the diagnosis of GGE and clearly documented myoclonic seizures as in some cases the treating physicians had not further subdivided the diagnosis of GGE.

### Antiepileptic Drug Outcome Classification

The effect of the antiepileptic drugs (AED) was evaluated retrospectively by detailed review of our medical records. Patients were included in the evaluation of the efficacy of a particular AED when they either became seizure free on the AED for ≥1 year independent of the dose or continued to have seizures despite reaching a minimal dose, which was defined as 600 mg/d for valproate (VPA), 1,000 mg/d for levetiracetam (LEV) and 100 mg/d for lamotrigine (LTG). Patients with insufficient data on the effect of the particular drug were not included. The number of exposed patients was insufficient to assess the efficacy of other AEDs.

Drug-resistance was defined by ILAE criteria (15), i.e., failure of adequate trials of two tolerated, appropriately chosen and used antiepileptic drug schedules in monotherapy or combination. Seizure freedom ≥1 year was considered as response. Patients, who were seizure free at last follow-up and drug-responsive, were assigned to group NDR (not drug-resistant, Figure 1). Patients, who were not seizure free at last follow-up and drug-resistant, were assigned to group DR (drug resistant, Figure 1).

### Identification of Misdiagnoses and Group Comparison

Medical records of group DR were reviewed in detail to identify patients who had been misdiagnosed as JME. In particular, seizure semiology, EEG, cerebral imaging, neurological examination, cognitive decline, psychiatric comorbidity and evolution of the epilepsy over time were considered. EEG reports in our center are standardized (16) and all reported features were taken into account. Patients were diagnosed with JME according to ILAE criteria (https://www.epilepsydiagnosis.org): (1) onset of MS and BTCS between 8 and 25 years and no other seizure types except for dialeptic seizures, (2) EEG with 3.5–6 Hz generalized spike-and-wave or polyspike-and-waves but no focal epileptiform discharges (except for fragments of generalized discharges), (3) no epileptogenic lesion on MRI, (4) normal neurological examination without evidence of ataxia or cognitive decline. Patients who only had myoclonic seizures on inappropriate AEDs were considered misdiagnoses. Mild intellectual disability and cognitive deficits without progressive decline were not considered exclusion criteria. To allow comparison of groups DR and NDR, medical records of both groups were reviewed regarding cognitive deficits, the presence of depression or psychosis at any time point, occurrence and frequency of DS and febrile seizures in the patients and first-degree relatives. To avoid assumptions on the type of epilepsy (focal vs. generalized) the semiological seizure classification was used (17). Based on the initial diagnosis of JME, one patient was included in the Epi25 Collaborative (18) and exome sequencing was done using blood DNA. The identified variant in *NHLRC1* was confirmed by Sanger sequencing. Statistical analyses were performed using IBM SPSS Statistics, Version 22.0 (IBM Corp., Armonk, NY, USA). Fisher's exact test was used to compare the parameters between groups DR and NDR. All *p*-values were two sided and regarded as statistically significant when <0.05.

## Results

Querying the electronic health information system for the search terms “myoclon”, “Myoklon”, or “Janz” identified 2,228 patients. Manual review revealed that 168 (74 male, 94 female) of these were diagnosed with JME at any time point (Figure 1). VPA was the most efficacious AED: 34/68 JME patients (50%) became seizure free for ≥1 year on VPA in monotherapy or combination as compared to 13/31 (42%) on LEV and 8/52 (15%) on LTG. Mean dose and standard deviation in seizure-free patients (dose in patients with ongoing seizures in brackets) was 1,228 ± 455 mg/d for VPA (1,526 ± 632 mg/d), 1,750 ± 650 mg/d for LEV (2,069 ± 935 mg/d) and 203 ± 71 mg/d for LTG (346 ± 144 mg/d). Eighty-seven of the 168 JME patients were seizure free at last follow-up including 61 drug-responsive patients (group NDR, Figure 1). Seventy-eight patients were not seizure free including 26 drug-resistant patients (group DR, Figure 1). Seizure frequency at last follow-up was unavailable in 3 patients.

Detailed review of the phenotype confirmed the JME diagnosis in 19 of 26 patients (73%) in group DR including 3 who only had myoclonus during the last year. The diagnosis was revised in 7 patients (27%, mean age 39 years, 57% females, Supplementary Table 1) including 6 patients in whom the JME diagnosis had already been questioned or revised during clinical follow-up. The revised diagnoses included 2 patients with GGE and drug-induced myoclonus only, 2 patients with JME and additional focal epilepsy, 1 developmental and epileptic encephalopathy, 1 focal epilepsy with secondary bilateral synchrony and 1 PME. The patient with PME was initially reviewed during the study at age 29 years and considered to resemble epilepsy with eyelid myoclonias (Jeavons Syndrome) but not PME due to pronounced eye-closure-induced polyspikes associated with eyelid myoclonia. However, she severely deteriorated at age 30 years with frequent BTCS, action-induced myoclonus and cognitive decline. Genetic testing revealed the diagnosis of Lafora disease due to a homozygous mutation in *NHLRC1* (c.G436A, p.D146N, NM_198586) whereas skin biopsy was unremarkable.

Comparison of the groups DR and NDR indicated that intellectual disability, cognitive impairment, febrile seizures in first-degree relatives and prominent DS were significantly more frequent in group DR (Table 1). Cognitive impairment remained more frequent in group DR when only deficits confirmed by neuropsychological testing were considered. After exclusion of the 7 misdiagnosed patients of group DR, the occurrence of DS at any time point became significant but intellectual disability was not significant anymore.

**Table 1:** Comparison of the groups DR (drug-resistant) and NDR (not drug-resistant).

**Parameter**	**Group NDR (*n* = 61)**	**Group DR**			
		**Including misdiagnoses (*****n*** **=** **26)**		**Excluding misdiagnoses (*****n*** **=** **19)**	
	**Number (%)**	**Number (%)**	***p*-value^a^**	**Number (%)**	***p*-value^a^**
**Sex (female)**	35 (57%)	12 (46%)	0.358	8 (42%)	0.297
**Intellectual disability**	0 (0%)	3 (12%)	**0.025**	2 (11%)	0.052
**Cognitive deficits**					
Reported by patient	10 (16%)	17 (65%)	**<0.001**	11 (58%)	**0.001**
Confirmed by neuropsychological testing	4 (7%)	13 (50%)	**<0.001**	9 (47%)	**<0.001**
**Depressive episode at any time point**	15 (25%)	12 (46%)	0.075	9 (47%)	0.085
**Psychosis at any time point**	0 (0%)	1 (4%)	0.299	1 (5%)	0.237
**Dialeptic seizures**					
At any time point	12 (20%)	11 (42%)	0.060	9 (47%)	**0.040**
As a prominent seizure type^b^	0 (0%)	4 (15%)	**0.009**	4 (21%)	**0.003**
**Febrile seizures**					
In patients	0 (0%)	2 (8%)	0.084	2 (11%)	0.056
In first-degree relatives	0 (0%)	3 (12%)	**0.023**	3 (16%)	**0.012**
**Seizure freedom for** **≥** **1 year on**					
Valproate^c^	28/42 (67%)	0/23 (0%)		0/17 (0%)	
Levetiracetam^d^	13/31 (42%)	0/14 (0%)		0/10 (0%)	
Lamotrigine^e^	7/26 (27%)	0/23 (0%)		0/16 (0%)	
Topiramate^f^	1/6 (17%)	0/9 (0%)		0/7 (0%)	

*Statistical tests were done comparing group NDR with total group DR and also comparing group NDR with group DR but excluding the patients who were finally considered to be misdiagnoses of JME*.

a Calculated with Fisher‘s exact test, significant values in bold;

b ≥1 dialeptic seizure/month at last follow-up and dialeptic seizures more frequent than other seizure types, denominator indicates the total number of patients exposed to a minimum dose of;

c 600 mg/d for valproate;

d 1,000 mg/d for levetiracetam;

e 100 mg/d for lamotrigine;

f *75 mg/d for topiramate*.

## Discussion

This retrospective analysis of our JME cohort revealed that 26/168 patients (15.5%) were not seizure free at last follow-up and drug resistant according to ILAE criteria. Detailed phenotypic review of these patients identified 7 misdiagnosed patients including one who was finally diagnosed with PME. Interestingly, this patient had been reviewed within the study one year earlier and was felt to be most consistent with a diagnosis of epilepsy with eyelid myoclonias but genetic testing confirmed Lafora disease.

Our findings confirm that individuals with PME but misdiagnosed as JME are found among drug-resistant patients in a tertiary epilepsy center. Even a very detailed review by experienced epileptologists may not identify the presence of PME before the typical features evolve. The mutation identified in our patient had been reported in the past to be associated with late onset and slow progress (19–22) emphasizing the need for early genetic testing in drug-resistant JME patients. Particularly with the prospective advent of personalized treatments in PME that may delay the progress of the disease, it is paramount to correctly diagnose the patients as early as possible.

Not only patients with Lafora disease but also with other types of PME may resemble JME at epilepsy onset. However, Mumoli et al. did not find *CSTB* repeat expansions in a cohort of 57 patients with confirmed JME diagnosis of which the majority (*n* = 48) was drug responsive (23). This suggests that the molecular analysis of *CSTB* is unlikely to be positive if patients are drug responsive and no atypical features are present.

In 6 of the 7 misdiagnosed patients the diagnosis of JME had already been questioned or revised during clinical follow-up. The presence of intellectual disability may suggest a developmental and epileptic encephalopathy. Two GGE patients had received a misdiagnosis of JME in stages when MS were induced by an inappropriate choice of drugs. These patients had not had MS before starting or after stopping the inappropriate medication. The differentiation between JME and focal epilepsy was an issue in three further patients. One case was considered to have only focal epilepsy with secondary bilateral synchrony on review whereas two further cases were diagnosed with JME and additional focal epilepsy. The latter showed a typical EEG pattern and seizure evolution for JME but had additional EEG or semiological findings consistent with focal epilepsy.

The frequency of drug resistance in our JME cohort (15.5%) was within the range reported in the literature (5–7, 9). It is well-known that VPA is the most efficacious drug in GGE (24) which was also evident in our study (50% seizure free for ≥1 year). Our data suggests LEV as an alternative (42% seizure free for ≥1 year) whereas LTG was poor (15% seizure free for ≥1 year). The high efficacy of VPA as compared to LTG is a particular issue for women in childbearing age in which VPA is avoided due to its teratogenic potential whereas LTG is preferred. However, we did not observe an increased frequency of females in group DR suggesting that this was not a major factor in our cohort.

Phenotypic features associated with a higher risk of drug resistance in our cohort even after excluding the misdiagnosed patients were the presence of cognitive impairment, febrile seizures in first-degree relatives and prominent DS. Our findings on cognitive impairment and DS are in line with previous studies (3, 25). In some of our cases, the patients and treating physicians judged the cognitive impairment to be related to the AED treatment whereas in others it was considered to be unrelated. The higher frequency of febrile seizures in first-degree relatives may suggest that genetic susceptibility factors for epilepsy also influence drug resistance. However, we cannot exclude an ascertainment bias as drug-resistant patients receive more frequent and detailed follow-up potentially leading to a higher chance that the presence of febrile seizures is documented.

A drawback of our study is that we did not review the diagnoses of the seizure-free and drug-responsive cases with JME. Therefore, we cannot exclude that some of them may have been misdiagnosed as having JME as well. However, the efficacy of the AED treatment in this cohort suggests that the presence of misdiagnosed patients with typically drug-resistant epilepsy syndromes is unlikely. Furthermore, the clinical consequences of a misdiagnosis are low if patients are seizure free. As only a subset of patients in this study had consented to genetic testing we could not perform exome sequencing in the other misdiagnosed patients.

In conclusion, our data highlights that detailed phenotyping should be performed in patients with alleged drug-resistant JME to exclude the presence of other epilepsy syndromes. Patients with PME misdiagnosed as JME are found in this cohort and may not be clinically discernible before the typical features evolve. This strongly underpins the need for early genetic testing if any features are present that challenge the diagnosis of JME.

## Data Availability

Data generated within this study will be made available on request. Data generated within Epi25 was deposited into dbGAP if participants provided consent for this.

## Ethics Statement

The local IRB, the ethics committee of the Department of Medicine, Philipps University Marburg, does not require extra ethical approval and patient consent, if data are collected retrospectively; any patients in our University Hospital agree upon admission that de-identified data might later be used for research purposes. The genetic studies were approved by the local IRB and the patient provided written informed consent.

## Author Contributions

SM and KMK conceived and designed the study. SM, SL, KK, AC, SK, and KMK collected the data. SM, AS, KK, PR, KM, AC, FR, SK, and KMK analyzed and interpreted the data. SM and KMK drafted the manuscript. AS, SL, KK, PR, KM, AC, FR, and SK revised the manuscript critically for important intellectual content. All authors approved the final version for submission.

### Conflict of Interest Statement

AS reports personal fees and grants from Desitin Arzneimittel, Eisai, LivaNova, Sage Therapeutics, UCB Pharma and Zogenix. PR reports reports personal from Eisai. KM reports consultancy fees from UCB and Eisai. FR reports personal fees from Eisai, grants and personal fees from UCB, grants and personal fees from Desitin Arzneimittel, personal fees and other from Novartis, personal fees from Medtronic, personal fees from Cerbomed, personal fees from ViroPharma, Sandoz, BayerVital and Shire, grants from the European Union, and grants from Deutsche Forschungsgemeinschaft. SK served on advisory boards for UCB and held lectures for UCB, Desitin and Eisai. She is study investigator for Sage, UCB and Boehringer Ingelheim, Marinus Pharmaceuticals Inc., Upsher-Smith Laboratories Inc., GlaxoSmithKline and Eisai Limited. KMK reports personal fees from UCB Pharma, Novartis Pharma AG, Eisai and GW Pharmaceuticals. The remaining authors declare that the research was conducted in the absence of any commercial or financial relationships that could be construed as a potential conflict of interest.

## Footnotes

^1^ILAE commission on classification and terminology. *Juvenile Myoclonic Epilepsy*. Available online at: https://www.epilepsydiagnosis.org/syndrome/jme-overview.html.
