# Supplementary material for: Drug-Resistant Juvenile Myoclonic Epilepsy: Misdiagnosis of Progressive Myoclonus Epilepsy
Source: Front Neurol. 2019 Sep 10;10:946. doi: 10.3389/fneur.2019.00946 (PMC6746890; doi:10.3389/fneur.2019.00946)
Supplement: Supplementary file 1 [file Table_1.pdf]

Supplementary table 1: Phenotypic details of the seven patients misdiagnosed as having juvenile myoclonic epilepsy

Patient	Seizure types (age of onset)	Cognition (age)	Neurological examination	Additional features	EEG	Brain imaging	Age at JME diagnosis	AED history and maximum dose	Revised diagnosis
1	Eyelid myoclonia induced by eye closure (onset unknown)<br>MS (17 yrs)<br>BTCS (20 yrs)<br>Worsening at 30 yrs with action-induced myoclonus, bilateral tonic seizures of the arms and BTCS after reducing VPA	Language and memory decline (30 yrs)[2]	Unremarkable (25+30 yrs)	Parents consanguineous, brother epilepsy and eyelid myoclonia, depression	GSWC 12 Hz, eye closure induced polyspikes, mild background slowing (25 yrs)<br>GSWC, bilateral MS, bilateral tonic seizures of the arms, moderate background slowing (VEM, 30 yrs)	MRI unremarkable (25 yrs)	25 yrs	VPA 2400 mg, LTG 225 mg, LEV 4000 mg, ZNS 100 mg, CLP 1 mg	Possible epilepsy with eyelid myoclonias, Jeavons Syndrome (29 yrs)<br>Lafora disease (30 yrs): *NHLRC1* (c.G436A, p.D146N, NM_198586)
2	DS (6 yrs)<br>MS (6 yrs)<br>BTCS (later)	ID (from early life)	VPA-induced tremor (38 yrs)<br>unremarkable (49 yrs)		GSWC 3-4 Hz, background slowing (34 yrs)	CT unremarkable (49 yrs)	34 yrs	CBZ 800 mg, VPA 2500 mg, LTG 300 mg, LEV (AE), TPM 250 mg, ZNS (AE)	Developmental and epileptic encephalopathy
3	Vertiginous and somatosensory aura (10 yrs)<br>BTCS (10 yrs)<br>MS (later)	Mnestic deficits (33 yrs)[3]	Unremarkable (27+35 yrs)	VNS implantation (27 yrs), sister epilepsy	GSWC 3-5 Hz, FPR 4°, right temporal SW, DS with GSWC → BTCS, no epileptiform activity during MS (VEM, 27 yrs)	MRI unremarkable (28+35 yrs)	24 yrs	ESM 1000 mg, ZNS 800 mg, CLB 15 mg, VPA 1500 mg, CBZ, OXC, LEV, TPM 150 mg, GBP, PHT, LTG, TGB, VGB, LCM, PER, BRV 50 mg	GGE and focal epilepsy

4	DS (13 yrs)<br>BTCS (13 yrs)<br>MS only on OXC and LTG (3rd decade)	None	Unremarkable (29 yr)<br>action and postural tremor (39+46 yrs)		DS with GSWC 3Hz (13 yrs)<br>GSWC, FPR (38 yrs)	MRI: superior vermis atrophy, otherwise unremarkable (55 yrs)	38 yrs	PRM 975 mg, VPA 1800 mg, LTG 550 mg, ESM 1000 mg, OXC 2400 mg, LEV 1500 mg	JAE with drug-induced myoclonus
5	MS (13 yrs)<br>BTCS (13 yrs)<br>Possible abdominal aura (onset unknown)	Mildly impaired divided attention and visuospatial memory deficits (52 yrs)[3]	Unremarkable (52 yrs)	Depression	Recurrent MS+reduced awareness with GSWC 2-3 Hz, continuous left temporal slowing, status epilepticus with focal EEG seizure pattern (VEM, 52 yrs)	MRI unremarkable (age unknown)	52 yrs[1]	LEV 4000 mg, LTG 650 mg, PB 50 mg, ESL 800 mg, TPM, ZNS 500 mg, VPA 1200 mg, CLB 5 mg	JME and focal epilepsy
6	Perioral MS (6 yrs)<br>MS predominantly right body (17 yrs)<br>BTCS (17 yrs)	Impaired divided attention (19 yrs)[3]	Unremarkable (18+19 yrs)	Depressive symptoms, borderline personality disorder, LZP abuse	GSWC and GPSWC, in 30% onset on the right, often associated with perioral MS, alpha and beta bursts with tonic contraction of lower face (VEM, 18 yrs)<br>GSWC (19 yrs)	MRI unremarkable (18 yrs)	18 yrs[1]	LTG 200 mg, LEV 3500 mg, VPA 2000 mg	Frontal lobe epilepsy with secondary bilateral synchrony
7	BTCS (17 yrs)<br>MS only on LTG/CBZ (32 yrs)	None	Unremarkable (41 yrs)	Polysubstance dependence	GPSWC, GSWC (32 yrs)	CT unremarkable (32 yrs)	32 yrs	CBZ 800 mg, LTG 400 mg, GBP, PB 350 mg, VPA (AE)	GGE with drug-induced myoclonus

[1] patient was diagnosed as GGE with documented myoclonic seizures at typical age for JME, [2] mental state assessment, [3] confirmed by neuropsychological assessment, AE: adverse event, BRV: brivaracetam, BTCS: bilateral tonic-clonic seizure, CBZ: carbamazepine, CLB: clobazam, CLP: clonazepam, DS: dialeptic seizure, ESL: eslicarbazepine, ESM: ethosuxiomide, FPR: fotoparoxysmal reaction, GBP: gabapentin, GGE: genetic generalized epilepsy, GPSWC: generalized poly-spike-wave complexes, GSWC: generalized spike-wave complexes, ID: intellectual disability, JAE: juvenile absence epilepsy, LCM: lacosamide, LEV: levetiracetam, LTG: lamotrigine, MS: myoclonic seizure, OXC: oxcarbazepine, PB: phenobarbital, PER: perampanel, PHT: phenytoin, PRM: primidone, TGB: tiagabine, TPM: topiramate, VEM: video-EEG monitoring, VGB: vigabatrin, VNS: vagal nerve stimulator, VPA: valproate, ZNS: zonisamide
